# Recovery of Recombinant Canine Distemper Virus That Expresses CPV-2a VP2: Uncovering the Mutation Profile of Recombinant Undergoing 50 Serial Passages *In Vitro*

**DOI:** 10.3389/fcimb.2021.770576

**Published:** 2022-01-14

**Authors:** Fuxiao Liu, Jiahui Lin, Qianqian Wang, Youming Zhang, Hu Shan

**Affiliations:** ^1^ College of Veterinary Medicine, Qingdao Agricultural University, Qingdao, China; ^2^ State Key Laboratory of Microbial Technology, Shandong University, Qingdao, China

**Keywords:** CDV, CPV-2a, reverse genetics, VP2 expression, next-generation sequencing, mutation

## Abstract

Canine distemper and canine parvoviral enteritis are infections caused by the canine distemper virus (CDV) and canine parvovirus type 2 (CPV-2), respectively. They are two common infectious diseases that cause high morbidity and mortality in affected dogs. Combination vaccines have been broadly used to protect dogs from infections of CDV, CPV-2, and other viruses. VP2 is the most abundant protein of the CPV-2 capsid. It elicits potent immunity in animals and, therefore, is widely used for designing subunit antigen-based vaccines. In this study, we rescued a recombinant CDV (QN vaccine strain) using reverse genetics. The recombinant CDV (rCDV-VP2) was demonstrated to express stably the VP2 in cells for at least 33 serial passages *in vitro*. Unfortunately, a nonsense mutation was initially identified in the VP2 open reading frame (ORF) at passage-34 (P34) and gradually became predominant in rCDV-VP2 quasispecies with passaging. Neither test strip detection nor indirect immunofluorescence assay demonstrated the expression of the VP2 at P50. The P50 rCDV-VP2 was subjected to next-generation sequencing, which totally identified 17 single-nucleotide variations (SNVs), consisting of 11 transitions and 6 transversions. Out of the 17 SNVs, 1 and 9 were identified as nonsense and missense mutations, respectively. Since the nonsense mutation arose in the VP2 ORF as early as P34, an earlier rCDV-VP2 progeny should be selected for the vaccination of animals in future experiments.

## Introduction

Canine distemper, caused by infection of canine distemper virus (CDV), is a highly contagious disease that affects a range of domestic and wild carnivores (McCarthy et al., 2007). Typical clinical signs include vomiting, diarrhea, dehydration, excessive salivation, coughing and/or labored breathing, loss of appetite, and weight loss (Liu et al., 2020b). Canine parvoviral enteritis, caused by infection of canine parvovirus type 2 (CPV-2), is another infectious disease that confers a common health problem in dogs. CPV-2-infected dogs show clinical signs within 3 to 7 days, presenting with severe gastroenteritis, lethargy, vomiting, fever, and diarrhea (Qi et al., 2020). Both canine distemper and canine parvoviral enteritis are relatively common in animal shelters and also are important issues of population management, as the immune status of incoming dogs is usually unknown (Litster et al., 2012). To date, combination vaccines have been broadly used for vaccinating dogs against CDV, CPV-2, and other viruses.

CDV is classified into the genus *Morbillivirus* in the family *Paramyxoviridae*, and its genome is a single strand of RNA with negative polarity, which encodes six structural (N, P, M, F, H, and L) and two non-structural (V and C) proteins in the order of 3′-N-P (V/C)-M-F-H-L-5′. Six open reading frames (ORFs) are separated by untranslated regions (UTRs) with variable lengths. The L protein is the RNA-dependent RNA polymerase (RdRp), lacking a proofreading mechanism in replication between viral genome and antigenome (Liu et al., 2016). Even so, CDV is still proven to be an effective vaccine vector to deliver foreign antigens in animals (Wang et al., 2012; Li et al., 2015; Miura et al., 2015).

CPV-2 is a non-enveloped, icosahedral, single-stranded DNA virus, belonging to the genus *Protoparvovirus* in the family *Parvoviridae*. The viral capsid contains 60 protein subunits of VP1 (5 to 6 copies) and VP2 (54 to 55 copies). The VP2 can be cleaved near its N-terminus by host proteases to produce another structural protein, VP3 (Miranda and Thompson, 2016). The VP2, the most abundant protein of the CPV-2 capsid, can induce robust immune responses in animals (López de Turiso et al., 1991; López de Turiso et al., 1992; Dahiya et al., 2012; Feng et al., 2014; Xu et al., 2014) and, thus, is the best subunit antigen conferring protective immunity in dogs. Now, CPV-2 is almost totally replaced by three antigenic variants, named CPV-2a, CPV-2b, and CPV-2c. The new CPV-2a has been epidemiologically predominant in recent years in China (Qi et al., 2020).

Sanger sequencing is unable to generate a large dataset to uncover a profile of viral quasispecies after serial passages. In other words, this conventional sequencing technique cannot be used for quantifying mutation rates of nucleotide sites in a viral genome. Next-generation sequencing (NGS), as an alternative method, has been successfully applied to analyze and to quantify the exceptionally high diversity within viral quasispecies. To date, a variety of viral genomes have been quantitatively analyzed by means of the NGS technique. A tremendous amount of NGS data is available in many public databases. Intrahost single-nucleotide variations (SNVs) have been systematically analyzed by the NGS to reveal evolutionary dynamics of numerous viruses (Campo et al., 2014; Hasing et al., 2016; Ni et al., 2016; Yang et al., 2018).

We had constructed a system of CDV (5804P strain) reverse genetics, whereby an enhanced green fluorescent protein (eGFP)-tagged CDV had been rescued from its cDNA clone. This recombinant virus had been subjected to serial passages *in vitro*, followed by NGS analysis to reveal quantitatively a mutated profile of the passage-47 (P47) progeny (Liu et al., 2021c). More recently, we have constructed another system of CDV reverse genetics based on a vaccine strain (QN strain) (Liu et al., 2021b). Using this system, we rescued here a recombinant CDV that could stably express the CPV-2a VP2 for at least 33 serial passages in cells. This VP2-tagged recombinant CDV (rCDV-VP2) was totally subjected to 50 serial passages *in vitro*, and then quantitatively analyzed through NGS for uncovering single-nucleotide mutations (SNMs) in its antigenome.

## Materials and Methods

### Cells, Virus, Plasmids, and Antibodies

Two cell lines, namely, T7 RNA polymerase-expressing BSR-T7/5 (Buchholz et al., 1999) and CDV infection-permissive Vero-Dog-SLAM (VDS) cell lines, were kindly provided by the China Animal Health and Epidemiology Center. Both were cultured at 37°C with 5% CO_2_ in Dulbecco’s modified Eagle’s medium (DMEM) supplemented with 10% fetal bovine serum and contained penicillin (100 U/ml), streptomycin (100 µg/ml), amphotericin B (0.25 µg/ml), and G418 (500 µg/ml). The wild-type CDV (wt-CDV), QN vaccine strain, was propagated in VDS cells. Three plasmids, pCAGGS-N, pCAGGS-P, and pCAGGS-L, had been constructed previously in our laboratory (Liu et al., 2020a) and would be used as helpers for virus rescue. CDV monoclonal antibody (MAb) was purchased from the Shandong Lvdu Bio-sciences & Technology Co., Ltd. (Lvdu, Binzhou, China). VP2 MAb was kindly provided by Dr. Chuanmei Zhang, Qingdao Agricultural University.

### Construction of rCDV-VP2 cDNA Clone

**Figure 1:**
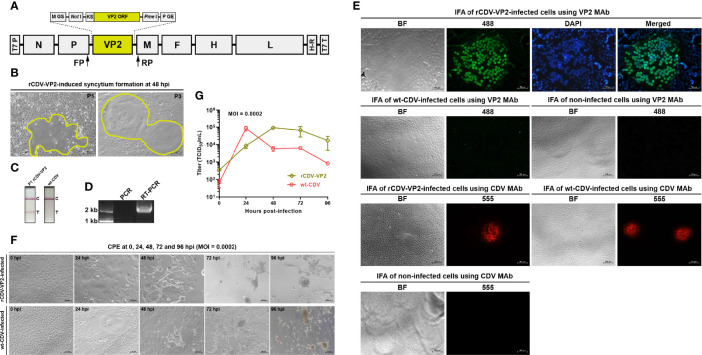
Rescue, identification, and characterization of rCDV-VP2. Schematic representation of rCDV-VP2 cDNA clone **(A)**. T7 P, T7 promoter; GS, gene start; GE, gene end; KS, Kozak sequence; H-R, hepatitis delta virus ribozyme; T7 T, T7 terminator. Syncytium formation on Vero-Dog-SLAM (VDS) cell monolayers during viral passaging **(B)**. Detection of rCDV-VP2- and wt-CDV-infected cell cultures by CPV-2 test strips **(C)**. T, test; C, control. RT-PCR analysis of P7 rCDV-VP2 using FP/RP **(D)**. The PCR assay is designed for exhibiting whether cDNA clone residues interfere with the RT-PCR analysis. Immunofluorescence assays (IFAs) of VP2 expression and rCDV-VP2 infection **(E)**. VP2 MAb (primary antibody) and Alexa Fluor^®^ 488 conjugate (secondary antibody) are used for the IFA of VP2 expression; CDV MAb (primary antibody) and Alexa Fluor^®^ 555 conjugate (secondary antibody) are used for the IFA of rCDV-VP2 infection. CPEs on VDS cell monolayers infected (MOI = 0.0002) either with the P15 rCDV-VP2 or with the wt-CDV at 0, 24, 48, 72, and 96 hpi **(F)**. Multistep growth curves of the P15 rCDV-VP2 and the wt-CDV within 96 hpi **(G)**. Data at each time point are representative of three independent experiments.

The CDV QN vaccine strain was previously subjected to next-generation sequencing, revealing a full-length sequence of viral antigenome, which was used for designing the rCDV-VP2 cDNA clone, schematically shown in **Figure 1A**. This cDNA clone was flanked by the T7 promoter and a hepatitis delta virus ribozyme-T7 terminator fusion sequence at its 5′ and 3′ ends, respectively. In order to improve its expression, the 1,755-bp-long ORF of CPV-2a (GenBank accession no.: JQ268283) VP2 was subjected to codon optimization based on *Sus scrofa*, and then flanked by the Kozak sequence (Kozak, 1987) at its 5′ end. The modified VP2 ORF (1,758 bp) harbored one extra “^4^GCG^6^” to meet the criteria of Kozak consensus sequence. The Kozak sequence-VP2 fusion fragment was flanked by *Not*I and *Pme*I recognition sites at its 5′ and 3′ ends, respectively. The modified VP2 ORF was regulated by M gene start (GS) and P gene end (GE) sequences at its 5′ and 3′ ends, respectively. The rCDV-VP2 cDNA clone was chemically synthesized in BGI Genomics Co., Ltd. (Shenzhen, China), subcloned into the pBR322 plasmid, and purified using the PureLink™ HiPure Plasmid Maxiprep Kit (Thermo Fischer, Carlsbad, USA) according to the instruction of the manufacturer.

### Rescue and Passaging of rCDV-VP2

BSR-T7/5 cells were seeded into a 12-well plate and cultured at 37°C with 5% CO_2_. A cell monolayer at 70% confluency was co-transfected with the rCDV-VP2 cDNA clone (2.0 µg/well), pCAGGS-N (1.0 µg/well), pCAGGS-P (0.5 µg/well), and pCAGGS-L (0.5 µg/well) using Lipofectamine 2000 (Thermo Fisher, Carlsbad, USA) according to the instruction of the manufacturer. The co-transfected cell monolayer was digested with trypsin at 72 h post-transfection (hpt), and then co-cultivated with VDS cells in a T25 flask. The rCDV-VP2 would be recovered, released from the BSR-T7/5 cells, and further infect the VDS cells. The rescued virus was subjected to serial blind passages in VDS cells.

### Test Strip Detection of VP2 Expression

The cell monolayer was subjected to three freeze-and-thaw cycles at 72 h post-inoculation (hpi) with the rescued rCDV-VP2. The supernatant was collected for a preliminary detection of VP2 expression by a CPV-2 test strip (Mensall^®^, Suqian, China), according to the instruction of the manufacturer. The wt-CDV-infected cell culture was also subjected to three freeze-and-thaw cycles for analysis as a control.

### RT-PCR Analysis of rCDV-VP2

The rCDV-VP2 was harvested at P7 for the extraction of viral RNA, which was used as template for RT-PCR analysis using the PrimeScript™ High Fidelity One-Step RT-PCR Kit (Takara, Dalian, China). The forward (FP: 5′-TCAAGAGTATTACTCATGCTTAA-3′) and reverse (RP: 5′-TCGAAGTCGTACACCTCAGTCAT-3′) primers targeted the P and M ORFs, respectively. RT-PCR reaction underwent 45°C for 10 min, 94°C for 2 min, and then 30 cycles at 98°C (10 s), 55°C (15 s), and 68°C (21 s). The extracted RNA was simultaneously subjected to PCR analysis as a control using the same primers. The PCR reaction contained 2× PrimeSTAR Max Premix (Takara, Dalian, China) and underwent 30 cycles at 98°C (10 s), 55°C (10 s), and 72°C (10 s). RT-PCR and PCR products were detected by agarose gel electrophoresis, followed by Sanger sequencing for analyzing the RT-PCR product.

### Indirect Immunofluorescence Assay of rCDV-VP2

VDS cell monolayers were independently infected with the P7 rCDV-VP2 and the wt-CDV for 24 h, and then fixed in 4% paraformaldehyde at room temperature for 30 min. After fixation, cells were washed four times with PBS, and then permeated with 0.4% Triton X-100 at room temperature for 30 min. After permeation, cells were washed three times with PBS and blocked in blocking solution at 37°C for 1 h. Subsequently, cell monolayers were independently incubated with the VP2 and CDV MAbs at 37°C for 2 h, followed by washing thrice with PBS. VP2 and CDV MAb-treated cell monolayers were incubated with the Alexa Fluor^®^ 488 and 555 conjugates (Thermo Fisher, Waltham, MA, USA) (1:250 in blocking solution) at 37°C for 1 h, respectively. Cells were washed three times with PBS, and VP2 MAb- and 488 conjugate-incubated ones were treated further by DAPI staining for 10 min. After coated with 90% glycerin, cell monolayers were observed under the fluorescence microscope. As controls, non-infected cell monolayers were subjected to the same treatments.

### Growth Kinetics of rCDV-VP2

VDS cells were seeded into five 12-well plates (10^6^ cells/well and 3 wells/plate) for incubation at 37°C for 2 h. The P15 rCDV-VP2 was inoculated (MOI = 0.0002) into all plates for incubation at 37°C for 3 h, and then the supernatants were replaced with DMEM for further incubation at 37°C. At 0, 24, 48, 72, and 96 hpi, any of the plates were randomly removed from the incubator and subjected to two freeze-and-thaw cycles to collect the supernatant for viral titration using the Spearman–Kärber equation (Finney, 1952). The wt-CDV as a control was subjected to the same treatments. The kinetic curve of virus growth was drawn using the GraphPad Prism software (Version 8.0). Data at each time point were representative of three independent experiments.

### Genetic Stability of VP2 During 50 Serial Passages

The rCDV-VP2 was serially passaged in VDS cells for 50 times (2–3 days/passage). The P7, 24, 29, 35, 41, 45, 46, 47, 48, 49, and 50 progenies were detected using the CPV-2 test strips, as described in the subheading *Test Strip Detection of VP2 Expression*. The P46, 47, 48, 49, and 50 progenies were additionally analyzed by indirect immunofluorescence assay (IFA) using the VP2 MAb as primary antibody, as described in the subheading *Indirect Immunofluorescence Assay of rCDV-VP2*. The P20, 30, 32, 33, 34, 36, 38, 40, 46, 47, 48, 49, and 50 progenies were subjected to RT-PCR assays as described in the subheading *RT-PCR Analysis of rCDV-VP2*, followed by Sanger sequencing.

### NGS of P50 Progeny

The P50 progeny was harvested for extracting total RNA, subsequently reverse transcribed by random hexamers using the 1st Strand cDNA Synthesis Kit (Takara, Dalian, China), according to the instruction of the manufacturer. The Illumina sequencing, library construction, and data processing were carried out at Shanghai Tanpu Biotechnology Co., Ltd. (Shanghai, China), as described in our previous report (Liu et al., 2021c).

## Results

### rCDV-VP2 Is Rescued from Its cDNA Clone

The BSR-T7/5 cell line was used for co-transfection of four plasmids to rescue the competent rCDV-VP2 from its cDNA clone. The VDS cell line was used for serial blind passages of rescued virus. The cytopathic effect (CPE), local cell-to-cell fusion, arose on the VDS cell monolayer as early as P1 (**Figure 1B**, P1). The typical syncytium formation was always visible during serial blind passages (**Figure 1B**, P3).

### Recovery of rCDV-VP2 Is Demonstrated by RT-PCR

The P7 rCDV-VP2 was analyzed by RT-PCR to confirm its identity. An expected band of amplicon size (2,037 bp) was observed only on the RT-PCR lane by agarose gel electrophoresis (**Figure 1D**, lane RT-PCR). As a control, PCR analysis (**Figure 1D**, lane PCR) showed no cDNA clone contamination affecting RT-PCR detection. The identity of rCDV-VP2 was confirmed by Sanger sequencing.

### VP2 Expression Is Demonstrated by Test Strip Detection and IFA

The rCDV-VP2- and wt-CDV-infected samples were detected by the CPV-2 test strips, indicating that the former could express the VP2 as early as P1 (**Figure 1C**). The IFA was carried out to confirm VP2 expression using the VP2 MAb and Alexa Fluor^®^ 488 conjugate as primary and secondary antibodies, respectively. Green syncytium formation was visible on the rCDV-VP2-infected cell monolayer. Moreover, DAPI staining suggested that the VP2 was mainly located in the nuclei (**Figure 1E**, the first panel). Neither wt-CDV- nor non-infected cells showed a similar cellular morphology (**Figure 1E**, the second panel). Additionally, the CDV MAb-based IFA confirmed that the rCDV-VP2 had been recovered from its cDNA clone (**Figure 1E**, the third panel).

### rCDV-VP2 Has Diverse Growth Kinetics from That of wt-CDV

To test the multistep growth curve of the rCDV-VP2 *in vitro*, VDS cells were infected with the P15 recombinant virus at MOI of 0.0002. Syncytia were observable at 24 hpi and exacerbated over time to cause intercellular hyperfusogenicity at 48, 72, and 96 hpi (**Figure 1F**, the first panel). The wt-CDV induced similar phenotypes on cell monolayers within 96 hpi (**Figure 1F**, the second panel). At each indicated time point, the virus-infected supernatant was collected and titrated in VDS cells. The growth curve of rCDV-VP2 was compared with that of the wt-CDV (**Figure 1G**). Both viruses exhibited different growth kinetics within 96 hpi. The rCDV-VP2 reached its peak titer (9.2 × 10^4^ TCID_50_/ml) at 48 hpi.

### One Nonsense Mutation Arises in VP2 ORF With Passaging

**Figure 2:**
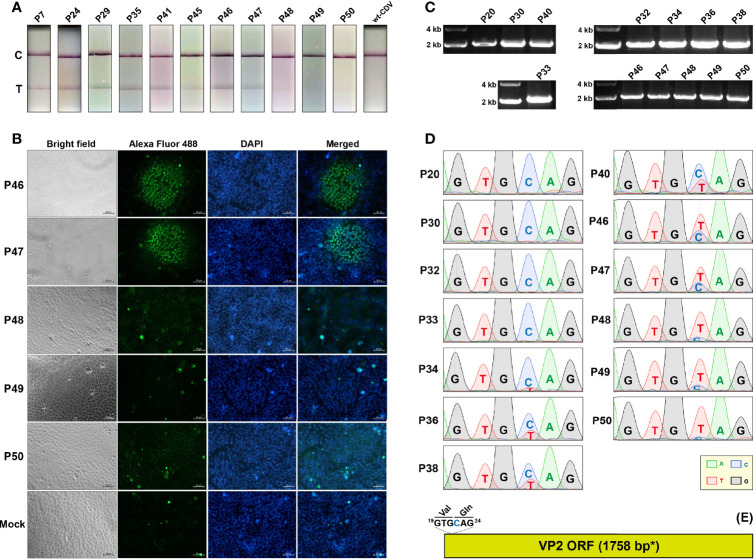
Genetic stability of VP2 sequence during 50 serial passages in VDS cells. Test strip-based detections of cell cultures independently inoculated with rCDV-VP2 progenies **(A)**. T, test; C, control. IFAs of VP2 expression in VDS cells inoculated with rCDV-VP2 progenies **(B)**. VP2 MAb (primary antibody) and Alexa Fluor^®^ 488 conjugate (secondary antibody) are used for the IFA. RT-PCR analyses of rCDV-VP2 progenies using FP/RP **(C)**. Sanger sequencing chromatograms of RT-PCR products of rCDV-VP2 progenies **(D)**. Only the 6-nt-long sequence, nt 19 to 24 in VP2 ORF, is shown. The ^19^GTGCAG^24^ fragment in VP2 ORF **(E)**. “GTG” and “CAG” encode valine and glutamine, respectively. *The VP2 ORF contains one extra “^4^GCG^6^” to meet the criteria of Kozak consensus sequence.

The rCDV-VP2 was serially passaged in VDS cells for 50 times. The P7, 24, 29, 35, 41, 45, 46, 47, 48, 49, and 50 progenies were detected using CPV-2 test strips. The results showed that the T (Test)-line was visible at P7 to 47, but nearly unobservable at P48 to 50 (**Figure 2A**). The IFA exhibited that, compared with the P46 and 47 progenies, the P48 to 50 did not effectively express the VP2 in VDS cells (**Figure 2B**). The RT-PCR analyses showed positive results for the P20, 30, 32, 33, 34, 36, 38, 40, 46, 47, 48, 49, and 50 progenies (**Figure 2C**), implying that the VP2 sequence was not deleted from the genomes of the progenies. All RT-PCR products were subjected to Sanger sequencing, which showed a point mutation, C22T, initially appearing in VP2 ORF approximately at P34, as evidenced by a double-peak (C/T) structure (**Figure 2D**, P34). At this site (**Figure 2E**, blue “C”), “C” was gradually replaced by “T” with serial passages, causing a stop codon TAG gradually becoming predominant in the VP2 ORF (**Figure 2D**, P34 to 50).

### NGS Uncovers Genomic Profile of the P50 Progeny

**Figure 3:**
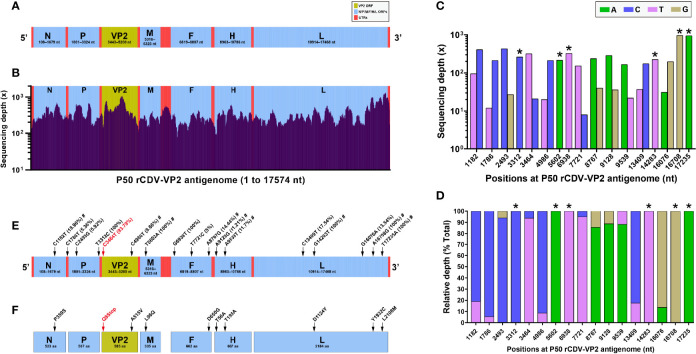
Next-generation sequencing of P50 rCDV-VP2. Schematic representation of rCDV-VP2 antigenome **(A)**. All elements proportionally match their actual lengths in the viral antigenome. UTR, untranslated region. Depth and coverage of NGS across the rCDV-VP2 antigenome **(B)**. All genetic elements proportionally match their actual lengths in the viral antigenome. Absolute **(C)** and relative **(D)** sequencing depths for identified sites with SNM. *Mutation rate of 100% at a given site. Profile of SNMs in rCDV-VP2 antigenome at P50 **(E)**. SNMs are marked with arrows. The sole nonsense mutation is marked with a red arrow. Numbers in brackets indicate point mutation rates. #Missense mutation. Amino acid mutations (arrow-marked) in seven proteins **(F)**. The sole nonsense mutation causes early termination of VP2 translation, marked with “Q8Stop” in red.

The rCDV-VP2 possesses a 17,574-nt-long genome. **Figure 3A** schematically shows all ORFs and UTRs in proportion to their actual distributions in the viral antigenome. The total RNA of the P50 progeny was extracted for NGS, which yielded analyzable sequencing depth and coverage (**Figure 3B**). The average sequencing depth was 352.5×, and the coverage range was approximately 99.9% across the full-length viral antigenome. Uncovered and low-depth sequences were only located at the 5′- and 3′-end regions in the antigenome. The NGS showed a total of 17 SNVs, consisting of 11 transitions and 6 transversions. **Figures 3C, D** show absolute and relative sequencing depths for all SNVs at P50, respectively.

The positions of SNVs were marked with arrows on the schematic representation of rCDV-VP2 antigenome (**Figure 3E**). Out of them, six positions (nt 3,312, 5,602, 6,938, 14,283, 16,708, and 17,235) yielded an estimated mutation rate of 100% (**Figure 3E**, bracket-marked). Only one UTR harbored a SNV (C1786T). There were a total of four low-frequency (less than 10%) mutations identified in the antigenome. The sole nonsense mutation, C3464T (**Figure 3E**, red arrow-marked), namely, the C22T in VP2 ORF (**Figures 2D, E** **)**, showed a mutation rate of 93.79%. Out of 17 SNVs, 9 were identified as missense mutations (**Figure 3E**, marked with “#”), accordingly causing 9 amino acid (aa) mutations (**Figure 3F**, black arrow-marked), distributed in the N, VP2, M, F, H, and L proteins.

## Discussion

Both CDV and CPV cause life-threatening infections with a major impact on the canine population (Vila Nova et al., 2018). Combination vaccines have been widely used against dog-susceptible viruses, such as CDV, CPV, rabies virus, canine parainfluenza virus, and canine adenovirus. CDV has been proven to be an ideal vector to express foreign proteins, such as fluorescence proteins (Plattet et al., 2004; Ludlow et al., 2012), luciferases (Parks et al., 2002; Liu et al., 2020a), and interleukins (Liu et al., 2014; Chen et al., 2019). CDV is also a promising vector for delivering foreign antigens in animals, therefore inducing efficient immune responses against canine distemper and other diseases (Wang et al., 2012; Li et al., 2015; Miura et al., 2015). The reverse genetics platform of the CDV QN strain was established previously in our laboratory. Using this platform, we have constructed many recombinant CDVs expressing foreign antigens, such as SARS-CoV-2 S1 protein (Liu et al., 2021b) and Dabie bandavirus Gn/Gc (data not shown). In the present study, the rCDV-VP2 was rescued from its cDNA clone using reverse genetics. The VP2, the most abundant protein of the CPV capsid, induces efficient immunity in hosts (López de Turiso et al., 1991; López de Turiso et al., 1992; Dahiya et al., 2012; Feng et al., 2014; Xu et al., 2014). We will attempt to explore the vaccine potential of rCDV-VP2 in eliciting immune responses against CDV and CPV in animal experiments.

The BSR-T7/5 cell line was used for co-transfection of four plasmids to rescue the recombinant virus, subsequently passaged in VDS cells. Based on our previous experience, morbillivirus infection-like CPE generally appeared at P2 or P3 (Liu et al., 2019a; Liu et al., 2019b; Liu et al., 2020a), whereas the rCDV-VP2 induced the typical CPE, syncytium formation, as early as P1, suggesting its robust adaptation to VDS cells. Extensive cell-to-cell fusion would reduce the release of morbilliviral progenies from the cell membrane (Cathomen et al., 1998; Liu et al., 2015), thereby unfavorable to high-titer virus replication in cells. In addition, the VP2 sequence, as a foreign gene or a genetic “burden,” would reduce the ability of viral propagation. Indeed, the rCDV-VP2 grew more slowly than the wt-CDV did within 24 hpi, whereas the former maintained a higher titer level than the latter did during 48 to 96 hpi.

The VP2 was successfully expressed by the rCDV-VP2 at P1, as evidenced by the detection of the CPV-2 test strip. It has been widely demonstrated that the VP2, if expressed in cells, can self-assemble into virus-like particles (VLPs) (Feng et al., 2014; Xu et al., 2014; Jin et al., 2016). We did not explore here whether the rCDV-VP2-expressed VP2 could self-assemble into VLPs in VDS cells. In a CPV-infected cell, the VP2 was expressed outside the nucleus, and then transported toward the nucleus for packaging of viral progenies (Tu et al., 2015). In this study, the IFA showed that the rCDV-VP2-expressed VP2 was primarily localized in the nucleus, as evidenced by a green (Alexa Fluor^®^ 488)-blue (DAPI) merged morphology in **Figure 1E** (the first panel). This was in contrast with another conclusion in a previous report, which displayed that nuclear transport of CPV VP2 in the assembled form was hampered in the absence of other viral components (Gilbert et al., 2005). Yuan and Parrish (2001), nevertheless, showed that the VP2, albeit expressed alone, could accumulate in the nucleus of the A72 cell (Yuan and Parrish, 2001).

As a non-self sequence of rCDV-VP2, the VP2 ORF harbored a nonsense mutation at nt 22 (at nt 3,464 in the viral antigenome), causing the early termination of translation at aa 8 in the VP2. This nonsense mutation was identifiable at P34 by Sanger sequencing. It initially showed a low frequency of occurrence, but became predominant in viral quasispecies at P50 (**Figure 2D**, P50), directly responsible for no clear T-line observable on the test strip (**Figure 2A**, P50). Because the VP2 was uninvolved in a series of CDV-related events, like replication, transcription, and packaging, any random SNVs would be theoretically retainable in the VP2 ORF with viral passaging. Unlike the VP2 ORF, viral self-genes underwent no lethal SNV with passaging (**Figure 3E**). Even though a given lethal mutation occurred in a viral self-gene, the mutated genome would not accumulate in a viral population, even rapidly eliminated with passaging to avoid the impact of error catastrophe on viral progenies.

Compared with most DNA viruses, morbilliviruses are characterized by the high mutation rate in their genomes with serial passages, mainly attributed to the lack of effective proofreading activities in their RdRps [see our previous review (Liu et al., 2016)]. The measles virus was estimated to have a mutation rate of 9 × 10^-5^/nt/replication and a genomic mutation rate of 1.43/replication (Schrag et al., 1999). More recently, Zhang et al. (2013) demonstrated that mutation rates in measles virus are the same for wild-type and laboratory-adapted viruses. The actual mutation rate for the measles virus is approximately 1.8 × 10^-6^/nt/replication event (Zhang et al., 2013). We recently found that the eGFP-tagged CDV (5804P strain), independently undergoing 40 extra passages in mutagen (ribavirin)- and non-treated VDS cells, exhibited 62 and 23 SNMs in viral antigenomes, respectively (Liu et al., 2021c). In this study, only 17 SNMs were identified in the rCDV-VP2 antigenome at P50. Under the same condition of non-selective pressure, the rCDV-VP2 as a negative-stranded RNA virus showed a significantly lower mutation rate than Senecavirus A (a positive-stranded RNA virus) did, as described in our previous report (Liu et al., 2021a).

Our aim in this study was to construct a bivalent vaccine candidate against CDV and CPV. Based on this aim, we hoped to rescue a recombinant CDV, characterized by the high-fidelity replication during serial passages. The high-fidelity feature would play an important role in developing live-attenuated CDV vaccines, since a virulence-attenuating CDV (Rockborn strain) was reported to revert back to a highly virulent form after serial passages in dogs (Appel, 1978). Additionally, the high-fidelity feature would ensure that the foreign antigen is stably expressed for inducing repeatedly immune responses *in vivo*. Unfortunately, one nonsense mutation arose in VP2 ORF approximately at P34, and moreover, such a mutated genotype was predominant in the P50 quasispecies. Therefore, an earlier rCDV-VP2 progeny, such as the P15, should be selected for use in future animal experiments.

## Data Availability Statement

The datasets presented in this study can be found in online repositories. The names of the repository/repositories and accession number(s) can be found in NCBI PRJNA782088.

## Author Contributions

FL conducted the experiments and wrote the manuscript. JL and QW performed the experimental works. YZ and HS provided the fundings. HS supervised the project. All authors contributed to the article and approved the submitted version.

## Conflict of Interest

The authors declare that the research was conducted in the absence of any commercial or financial relationships that could be construed as a potential conflict of interest.

## Publisher’s Note

All claims expressed in this article are solely those of the authors and do not necessarily represent those of their affiliated organizations, or those of the publisher, the editors and the reviewers. Any product that may be evaluated in this article, or claim that may be made by its manufacturer, is not guaranteed or endorsed by the publisher.
